# Pathology and Practice *(Medical)*

**Published:** 1818-05

**Authors:** 


### PART III.

### MONTHLY SKETCH OF FOREIGN MEDICAL SCIENCE.

——— Sparsa Colligens.

### I. PATHOLOGY AND PRACTICE (*Medical*).

1. *Convulsive Diseases.* The interesting researches of Dr. Sanders upon the pathology of this class of diseases, have been more than once noticed by us in a tone of earnestness and of eulogy, which they are well calculated to call forth from men zealously devoted to the interests of their science.* And scarcely a day passes by, in which we do not glean, either from observation or reading, some fresh evidence, collateral or direct, favourable to the correctness of the new doctrines, and to the revolution in medical opinions and practice, which they are destined, ere long, to accomplish. Truth must ultimately triumph over all the obstacles which impede her progress; and there never, perhaps, was a period in the history of human affairs, when the mind evinced so strong a tendency to

* See the Selections in the *Medico-Chirurgical Journal* for last November.

revolt against the dominion of prejudice, and raise itself from the despotism of long-received authority, as the present. We, ourselves, were at first startled by the boldness and novelty of Dr. Sanders's views and assertions, and received them with a suspicion, and criticized them with a severity, which nothing but a painful experience of the general emptiness of medical theories could have inspired or justified. This erroneous judgment we have long since endeavoured to expiate: and, notwithstanding the obloquy and ridicule which some learned gentlemen have affected, and still affect to cast upon "the new-fangled doctrines of the spinal school," we confidently predict, that those doctrines will ultimately acquire a general prevalence and establishment; and, that by the medical historian of future times, the name of their author will be registered on the same page with those of the distinguished men, whose genius and labours have most contributed to the improvement and dignity of the science.

With these prefatory remarks, we shall introduce to the notice of our readers an Extract from a Memoir on Epilepsy, by Dr. Esquirols,* very nearly bearing upon, if not directly illustrating, the doctrines in question. We have no doubt but that, in the event of its attracting the notice of Dr. Sanders, it will be perused by him, and all who correctly estimate the originality and importance of his researches, with peculiar interest.

Case 1. A young woman, who had been epileptic from her twenty-eighth year, became maniacal, at thirty-three, in the intervals of the epileptic paroxysms; and died at the age of thirty-seven, after the occurrence of several fits in rapid succession. On Dissection, the spinal marrow was found softened towards the eleventh and twelfth dorsal vertebræ. The softened medullary substance displayed a slightly brown colour.

Case 2. A woman, who had long been epileptic, contracted syphilis at the age of thirty-one. By the copious employment of mercury, the return of the fits was accelerated; and mania came on, sometimes before, sometimes after, the epileptic paroxysm. The woman died, in her thirty-seventh year, from an organic lesion of the uterus

---

* *Extrait d'un Mémoire du Docteur Esquirols sur l'Epilepsie, &c. Bulletin de la Faculté de Médecine de Paris, &c,* 1817, No. vi.

and ovaria. The lumbar extremity of the spinal marrow exhibited an increase of density in comparison with the other portion of the medullary substance.

Case 3. ———— had been epileptic from infancy, and experienced daily attacks of epileptic vertigo. She was very irascible, and after the fits attempted suicide. She, at length, died from asphyxia during an epileptic paroxysm. The meninges were injected (with blood), and the spinal marrow softened at its lumbar extremity.

Case 4. A girl, who had become epileptic at the age of thirteen, in consequence of fright, was often in a state of dementia after the paroxysm. She was sometimes furious, and frequently suffered from vertigo. She died about her nineteenth year, after several epileptic fits rapidly succeeding each other. A point of suppuration was found in the white substance of the posterior part of the left lobe of the brain. The arachnoid covering of the spinal marrow exhibited towards the tenth and twelfth dorsal vertebræ, a brownish appearance; and, in the two corresponding points, the medullary substance was softened.

Case 5. A girl, epileptic from birth, in consequence of a fright sustained by her mother during pregnancy, had fits every fortnight till the age of twenty. After one of these, she lay for a month in a lethargic state. In the ensuing spring, she remained one fortnight in a similar condition, and died after a violent epileptic paroxysm. The arteries of the basis cranii were, in several points, cartilaginous and ossified. The parietes of the aortic ventricle of the heart were an inch in thickness, while the cavity itself measured but from five to six lines. Towards the eleventh and twelfth dorsal vertebræ, the sheath of the spine (méningine) was brownish, and the medullary substance softened to the right and left.

Case 6. A female cook, became epileptic at the age of thirty-five, remained melancholy for two or three days after each paroxysm. She died asphyxiated during a fit about her fortieth year. The whole external surface of the spinal membranes was studded with osseous scales of a dull white colour, and from one to two lines in diameter. Towards the seventh and eighth dorsal vertebræ, and the lumbar extremity of the spinal marrow, the medullary substance was softened.

Case 7. A woman remained epileptic after convulsions induced by fright, at the age of fifty-three. The paroxysms recurred every second or third day, and were very violent at her fifty-sixth year. For several months, the fits recurred with increased frequency; and she died after a paroxysm which had left her for five days in a comatose state. Hydatids of various sizes were seen, from the bulb of the brain to the lumbar extremity of the vertebral canal, contained in the sac formed by the spinal membrane. The medullary substance was softened at its lumbar extremity. In the pituitary gland was enclosed a cyst filled with a reddish brown fluid.

CASE 8. A child had convulsions from the period of his first dentition. They afterwards degenerated into epileptic fits. At the age of four, the paroxysms increased in frequency. A year and a half afterwards, he had four or five fits a day, and became paralytic. Between the sixth and seventh year he died. The spinal membrane was gorged with blood; and the medullary substance exhibited a yellowish colour, and was softened in the vicinity of the sixth and twelfth dorsal vertebræ.—Dr. Esquirols observes, that the preceding eight cases have not been selected from others in order to establish "a new theory." From the first of February to the first of June 1817, ten epileptic patients died at *La Salpetriere*. The bodies of nine of these were submitted to examination; and in seven of them, were discovered lesions of the spinal marrow, or its membranous coverings.

In *one* case, the meninges were gorged with blood; in *two*, the arachnoid covering of the spinal marrow was greyish; in *one*, it was studded with depositions of bone. In *one*, there were hydatids in the whole of the sac formed by the spinal membrane from the bulb of the brain to the lumbar extremity of the spinal canal. The medullary substance of the spinal marrow was softened in different portions of its extent, most frequently at the lumbar extremity of the organ. In *two* instances, the softening was less decided; and in *two*, the softened portions exhibited a change of colour.

From these facts, may it not be inferred, that there are some epilepsies dependent on lesions of the spinal marrow or its membranous coverings? Dr. Esquirols thinks, that on comparing the influence of the spinal nerves upon the functions of locomotion with the disturbed state of the locomotive system during the epileptic paroxysm, we shall be more disposed to admit this variety of the disease. These considerations led him to an inspection of the spine of epileptics; and the event has justified his suspicions.

Directed by this inference, he applied four moxas along the spine of an epileptic patient. The disease had commenced at the age of sixteen. The paroxysms recurred every eight or ten days, and invariably at the menstrual period. At that time, she had four or five fits in a day. The moxa was first applied on the 22d of April; and, thenceforth, the young woman had but three fits. Menstruation had twice passed over without any paroxysm.

This case, however, is not conclusive; for the success of the treatment was incomplete: and, had it been otherwise, a solitary fact proves nothing. In publishing it, and the result of his dissections, Dr. Esquirols wishes to direct the attention of pathologists to an object of research, in his opinion the more interesting, as epilepsy is a disease, upon the seat of which we possess very few positive data (le moins de données positives).*

We scarcely need remark how much the value of this communication is enhanced, by its being the production of a foreigner, who, as may be obviously inferred from his silence, is unacquainted with the peculiar views and opinions of Dr. Sanders; and, consequently, cannot be interested in their propagation and support. Evidence, like this, cannot be without effect even on the most stubborn sceptic.

---

### *Hydrophobia.*

Much discussion has recently taken place among the medical writers of the continent upon the important question whether the *disease, induced by the bite of a rabid animal,* be attributable to the operation of a specific poison secreted in the salivary glands of such animal, and instilled into the wound at the moment of its infliction, or whether the symptoms may be simply considered as the result of a high degree of nervous irritation, arising from the injury, or the state of mental disquietude and alarm connected with it.† The failure of every attempt to communicate hydrophobia to healthy dogs, by inoculation with the supposed virus, and the fact that all the characteristic symptoms of this dreadful malady have been known to ensue from the wound inflicted by otherwise healthy animals, in a *transient* paroxysm of irritation, are

* We give the orignal here, as, in a valuable contemporary Journal, the sense of it has been here, as in some other parts, strangely perverted by the Translator.

† See *New Medical and Physical Journal,* vol. ix. p. 508.

supposed to substantiate the latter inference. It now, however, seems to be pretty generally admitted that genuine hydrophobia invariably originates from the application of some specific but unknown virus to an abraded portion of surface of the animal organs. Still, we are far from being unwilling to allow that cases may occasionally occur, wherein all the morbid phænomena exhibited by the hydrophobic, are rather referrible to the physical or moral disturbance excited by the wound, than to the influence of any peculiar or virulent property which has been acquired by the saliva deposited in it. How far this view of the subject may tend to settle the controversy in which it is at present involved, and to explain the glaring discrepancies of observation and experience in the medical history and treatment of hydrophobia, we presume not to determine. Confident we feel that such view is as correct as it is impartial; nor can we adduce a more unquestionable proof of its soundness, than that which we are about to transcribe from a respectable Italian Journal.*

A man, aged twenty-three, in attempting to separate his dog from another with which it was furiously contending, on the 26th of June, 1816, received a bite in the leg. The wound, however, was so slight as not to attract notice; and, in two days, it was cicatrized. On the 30th, the dog, from which the injury had been received, was no where to be found. Immediately the young man became dejected, and his mind haunted by the apprehension that the animal must have been mad. By the failure of reiterated search for the dog, the poor fellow's distress was, on the morrow, aggravated. He began to avoid the friends who came to offer consolation, and openly expressed his fears respecting the nature of his malady. On the same day, he evinced horror of water and other slight hydrophobic symptoms; and would not, or could not, swallow any liquid. In the evening, on being imprudently told that some persons in the neighbourhood had been bitten by a mad dog, he burst into tears, and ordered every one to quit his chamber.

On the 2d of July, the 6th day from the reception of the wound, some people, attracted by an uproar in the house, found the young man brandishing a musket in a state of furious agitation, and on the point of sallying forth. He,

* *Giornale di Fisica*, Jan. and Feb. 1817.

however, was restrained and carried to bed. The surgeon of the place was not recognized by the patient, and he inquired with terror who the stranger was.

After a short interval of profound dejection, the young man was seized with violent paroxysms of fury, such as are commonly observed in hydrophobic patients; and it became necessary to secure him. This state lasted two days, during which he obstinately rejected all aliment, solid and liquid, and could not even be prevailed upon to take a little water. On the morning of the 5th, the condition of the patient still remaining unchanged, the suspected dog was fortunately found. No time was lost in communicating to him this intelligence; but he, at first, refused to credit it. When convinced, he requested that he might once more see his dog before he died. The animal, on being brought into the chamber, leaped upon his master's bed, and fondled him in the accustomed manner. Meanwhile, the young man gradually recovered his serenity, and became quite tranquil. After the lapse of four days, he was perfectly restored to health.

It can, we think, scarcely admit of a doubt, that, had the dog in this instance never been recovered, or his return been delayed for some days longer, the unfortunate master must have inevitably fallen a victim to a purely moral affection simulating all the characters of genuine or communicated hydrophobia. This fact places, in a very conspicuous point of view, the impolicy of the vulgar practice of destroying dogs suspected of suffering from rabies, instead of fairly determining the question by vigilant observation of the phænomena and issue of the malady.*

In the preceding case, we cannot help remarking, that no allusion whatever is made to the state of the limb, upon which the injury had been inflicted, either immediately before, or during the invasion of the hydrophobic symptoms. The obvious inference is, that no sensible alteration had taken place in it. Now, we believe, that, in almost every instance of hydrophobia unquestionably resulting from the bite of a rabid animal, which has yet been recorded, some sensations of uneasiness about the site of the original injury, or traces of inflammation in the course of the absorbent vessels, will generally be

---

* It, moreover, frequently happens, that a suspected animal, although really healthy, or suffering some other disease, is goaded to acts of fury by the barbarity of his unrelenting pursuers. EDIT.

found to have preceded the developement of the first hydrophobic paroxysm: and, if this difference of condition of the local injury do, as we believe, really exist, might it not constitute a sufficiently correct criterion for diagnosis between genuine and spurious hydrophobia—between the disease induced by inoculation with a peculiar morbid poison, and that consequent on simple irritation propagated from the seat of injury to the brain, or excited in the system through the medium of a heated and distempered imagination.

---

### *Case of Hydrothorax, with Remarks.* By JOHN N. TAULMAN, A. B. of Rockland County, New York.*

THE following case occurred in the practice of the writer of this paper, at the New York Alms-house. The subject was a man of rather delicate habit of body, about 35 years of age, and in whom the sanguine temperament is strongly marked. His former occupation was that of a labourer, and he then was in the habit of using spirituous liquors. When dropsical symptoms first appeared, he had just completed a mercurial course, under which he was placed for the cure of secondary symptoms of syphilis. He was first infected with lues venerea, and had been repeatedly salivated: the principal part of that time he has been in this institution.

About the 10th of May, 1813, he observed some swelling of his legs: of this, however, as it gave him but little pain, he made no complaint. He had previously observed that his urine had become very much diminished in quantity. In this, he says, he cannot be deceived, so singular was the phænomenon. About three months ago he found that he passed but three or four ounces in the morning, and that with considerable difficulty; as much more was usually voided during the remainder of the day. Things remained in this state till symptoms of hydrothorax appeared.

The œdematous state of his lower extremities had not existed many days, before, as he relates, he discovered some difficulty in breathing after he ascended the stairs. His nights were restless; no sooner did he get into a sound sleep than he was suddenly awakened by a sense of anxiety and difficult breathing, from which he got no relief but by sitting up in bed.

---

* American Medical and Philosophical Register.

On the 24th of May, his situation became alarming. I found him sitting up, breathing with the greatest anxiety, in which manner he passed the preceding night. He had made several attempts to lie down, but was every time seized with a sense of suffocation, which obliged him to seek the erect posture. His pulse was remarkably full and strong, but not more frequent than natural; his respiration very much hurried, and inspiration short. From these symptoms, there was no hesitation in pronouncing his disease effusion in the cavity of the chest, the effect of increased arterial action; and, accordingly, he was immediately let blood to the amount of oz xxiv. The oppression diminished while the blood was flowing. A large dose of Glauber's salts was prescribed, which operated kindly. The next morning we found him considerably relieved; he had passed a comfortable night, and altho' his pulse had become more full and strong, yet further evacuations by the lancet were considered no way necessary. He was directed to take, to day, three doses of nitre, each grs. xv.; to abstain from animal food; and to remain quiet. This evening his symptoms returned, but he neglected to call for assistance till early on the morning of the 26th, when his condition was greatly aggravated. He was sitting on his bed, clenching with his hands the head of another bed, and leaning forward with all his weight, thus throwing into action every muscle that could possibly assist in respiration. The lancet was again resorted to, and he lost blood to the amount of oz. xvi; more blood would have been drawn, for very little alleviation of his distress took place, but the pulse fell considerably. A large blister was immediately applied to his chest, and he was directed to take of the tinct. digitalis, gtt. xl. In the afternoon, Dr. Gilbert Smith, the visiting physician to the Institution, was requested to see him. The patient had derived no benefit from the prescriptions of the morning, but in every respect seemed worse; the oppression under which he laboured was extremely distressing. Dr. Smith advised a repetition of blood-letting as the only remedy affording the least hopes of success; a vein was accordingly opened, and he was bled oz. xxxiv. He was considerably relieved during the flowing of the blood; pulsation became smaller and weaker, and he was able to rest in a semi-horizontal posture: a dose of Glauber's salts was exhibited. His legs having now become very much enlarged, it was conceived proper to evacuate by puncture the fluid collected in the cellular membrane.

27th. He passed a comfortable night in a semi-horizontal posture. The sal. Glauberi has produced copious evacuations. The quantity of fluid discharged from his legs is very great. He was now put on small doses of calomel and squills, for the purpose of exciting the various secretions, and particularly that by the kidnies.

28th. He slept soundly, his head not more elevated than usual; breathes naturally; takes a deep inspiration with ease: his pulse has become more frequent and full. He occasionally coughs, and expectorates, with ease, considerable bluish mucus. His urine is increased to double its late quantity. In addition to the calomel and squills, he was directed to take, every two or three days, a dose of Glauber's salts.

Notwithstanding these depleting remedies, and abstinence from every thing stimulating, his pulse continued to rise, respiration became more difficult, and the sense of suffocation, in a horizontal posture, in some measure returned. On this account it was thought prudent again to take blood from his arm, to avert the danger with which he seemed to be threatened; and, accordingly, on the 5th of June, he was bled oz. x; the pulse was reduced to its natural standard, and all sense of straightness of the chest entirely removed. He was directed to continue his remedies, with the addition of the spirit. æther. nitros. to excite more powerfully the secretion by the kidnies.

The œdematous state of his lower extremities has been nearly reduced by the continual discharge of fluid from the punctures. A roller was now applied, and he was directed to bathe his legs frequently in cold water.

Such a strong tendency was there to a return of his disease, that it was found necessary to continue this course till the 25th of June, before he could be considered as secure from a relapse.

June 25. His strength is greatly impaired, he is much emaciated, and the plethora of his blood vessels is diminished. Respiration is natural; cough and expectoration have ceased. He lies down without any inconvenience, and sleeps soundly. The quantity of urine has become natural, and is voided with ease. His legs are restored to their natural dimensions. He was now put on tonic remedies; ginger and the rust of iron were prescribed in doses of grs. viii each, to be repeated three times a day. He was also directed to drink frequently of the bitter infusion composed of quassia and gentian, with the addition of rhubarb ; to continue the local cold bathing, and to return gradually to his accustomed diet and exercise.

July 10. He is improving rapidly under the tonic treatment. No symptoms of his disease have returned.

The inordinate proportion of fluids which must have existed in this case, cannot be referred to the ingesta as a cause. On the contrary, being excluded from all spirituous and fermented liquors, and indeed from every thing that could have a tendency to fill his system, and having for a long time before the attack almost made mercury his food, we ought, à priori, to expect to find him then less robust than he formerly was ; and such actually was the case. The cause, then, of this fulness of the vessels is to be looked for elsewhere. It was before stated, that an interruption in the urinary secretion had existed for some months previous to his illness. Less fluid than natural being secreted by the kidnies, accumulation must have been the consequence, were there no other outlet for the fluids of the system ; and evidence of none existed, for his skin appears to have regularly performed its functions.

The warmth of the spring expanding the fluids, already distending the vessels by their quantity, probably acted as an exciting cause. Such, then, being ascertained to be the nature and causes of the disease, the indications of cure were very evident, and led to that free use of the lancet, and other evacuants, called for in all diseases arising from an excessive quantity of fluids.

This case shows the importance and necessity of that distinction in the causes of dropsies, which the books on practice do not generally make, and sufficiently enforce, and which we have never heard more clearly elucidated, than from the practical chair in the college of physicians and surgeons. In this remark we do not intend to include our late distinguished countryman, Dr. Rush, who has happily discriminated between the different causes of dropsies. Thomas, our latest systematic writer, mentions but one cause of dropsy, debility; and, of course, has but the one mode of treatment. Dr. Cullen, although he enumerates increased effusion among the causes of dropsies, yet, like that of Thomas, his treatment is calculated only for that kind which is the result of debility. Maclean, a late writer on hydrothorax, observes, that dropsy may arise either from increased exhalation, or from diminished absorption, or from both. But when he comes to investigate the manner in which this " increased exhalation" is produced, he dwells exclusively on relaxation of the solids concurring with tenuity of the blood, and thus makes dropsy the effect only of debility showing itself particularly in the exhalents and in the absorbents. But we would observe, that dropsies, and more particularly dropsy of the chest, frequently are consequent upon a plethora of the vessels, and a vigorous circulation; that dropsy does not always arise from a laxity of the exhalents, and a torpor of the absorbents. The extent to which blood-letting was here carried, may, perhaps, at first, seem unadvised and even empirical, but, upon more mature consideration, we trust, will be found fully justified by the nature and causes of the disease.

The grand remedy of Maclean, and on which he places his chief reliance, is the digitalis. By this, in combination with other medicines, he states, that he cured several cases of well marked hydrothorax, and relieved many more. He gives the treatment and termination of above eighty cases, and although he employed venæsection in but one, (in which he afterwards regrets that he did not employ it earlier and more extensively) yet in the body of his work, he does not seem unfriendly to this remedy. He observes, that, "to lay down venæsection among the remedies for the cure of this disease may appear inconsistent with the general principles held out; but however this may be, cases will sometimes occur where it may be absolutely necessary, and where safety, nay, *even the life of the patient*, may depend on its timely use; nor will it be found contradictory, on due consideration, to the general plan recommended." He, however, thinks that "very few hydropics will bear the loss of much blood."

With respect to the disease from which our patient had just recovered, when attacked with this more dangerous affection, I would observe, that it was removed by the use of the corrosive sublimate, with the addition of that valuable auxiliary, the decoction of the woods. This form of mercury, which, a few years since, was recommended in an Inaugural Dissertation on Mercury, by John W. Francis, as a safe, efficacious, and convenient remedy in the cure of lues venerea, has lately been almost exclusively employed in the alms-house, in all forms of the disease, and with uniform success.

